# Perioperative Antibiotic Prophylaxis in Cesarean Section and the Maternal Gut Microbiome: Protocol for a Remote Observational Cohort Study

**DOI:** 10.2196/84909

**Published:** 2026-04-22

**Authors:** Elisabeth AL Feles, Frauke Mattner

**Affiliations:** 1 Department of Human Medicine, Faculty of Health, Witten/Herdecke University, Alfred-Herrhausen-Strasse 50, Witten, 58455, Germany, 49 221-8907 ext 8677, 49 211-8907-8314; 2 Institute of Hygiene, University Hospital of Witten/Herdecke, Cologne Merheim Medical Centre, Cologne, Germany; 3 Central Pharmacy, University Hospital of Witten/Herdecke, Cologne Merheim Medical Centre, Cologne, Germany

**Keywords:** maternal health, peripartum health, cesarean section, CS, perioperative antibiotic prophylaxis, PAP, maternal gut microbiome, decentralized clinical research, remote observational study

## Abstract

**Background:**

Cesarean section (CS) requires perioperative antibiotic prophylaxis (PAP) for the prevention of surgical site infections. However, systemic antibiotics during the peripartum period may induce compositional perturbations of the maternal gut microbiome, a system already characterized by reduced resilience. Data on maternal gut microbiome dynamics after CS with PAP are scarce, largely due to logistical and feasibility barriers that limit the participation of pregnant women and new mothers in conventional clinical studies.

**Objective:**

This protocol primarily aims to evaluate the feasibility of a fully decentralized, remote study design for longitudinal gut microbiome research in the peripartum period. Secondary exploratory objectives include the comparative analyses of microbiome composition between CS with PAP and vaginal delivery (VD) without antibiotic exposure to inform future adequately powered studies.

**Methods:**

The MAMA (Microbiome Changes Due to Antibiotic Prophylaxis in Mothers at Birth) study is a prospective, 2-arm observational cohort study conducted entirely off-site. Women in the third trimester of pregnancy were recruited at 2 German level-1 perinatal centers and affiliated outpatient facilities. Participants underwent either CS with PAP (single dose cefuroxime 1.5 g intravenously) or VD without antibiotics. Stool samples were self-collected at home and returned by mail at 3 predefined time points: late pregnancy (T0), 2 to 3 days post partum (T1), and 90±10 days post partum (T2). Primary outcomes are feasibility indicators, including recruitment rate, sample and questionnaire return rates at each time point, adherence to sampling windows, and participant retention across follow-up. Secondary outcomes are exploratory microbiome measures based on 16S rRNA gene sequencing (V3-V4), including alpha diversity indices, beta diversity metrics, and relative taxonomic abundances. Microbiome analyses are explicitly compositional and hypothesis-generating. Group comparisons and longitudinal within-individual changes will be assessed using nonparametric diversity metrics and multivariate distance–based methods. No confirmatory hypothesis testing is planned.

**Results:**

Recruitment occurred between May 2022 and October 2023, with 37 women enrolled (25 CSs and 12 VDs). Follow-up was completed with receipt of the final stool sample in March 2024. DNA extraction and sequencing were completed in a single batch in October 2024. Bioinformatic processing and statistical analyses were initiated in June 2025 and are ongoing as of December 2025. Results from the exploratory microbiome analyses are expected to be published in 2026.

**Conclusions:**

This protocol demonstrates the feasibility of conducting fully decentralized, longitudinal microbiome research in a peripartum population without requiring on-site visits. By integrating study procedures into maternal realities, the remote design reduces participation barriers and addresses a clinically relevant research gap that has remained largely unexamined despite routine use of PAP. While microbiome-related outcomes are exploratory, the methodological framework established here provides a scalable model for future maternal and postpartum research, supporting ethically grounded, participant-centered study designs and evidence-informed care strategies.

## Introduction

### Background

Cesarean section (CS) accounts for a substantial and steadily increasing proportion of births worldwide (21% in 2021), representing one of the most common surgical interventions performed in otherwise healthy women [1]. Nevertheless, it carries the third-highest incidence of surgical site infections among all surgical interventions [2]. To reduce postoperative infectious morbidity, perioperative antibiotic prophylaxis (PAP) is universally recommended and constitutes a central component of obstetric surgical safety, as endorsed by major gynecological societies and supported by World Health Organization (WHO) guidelines [3-5]. Recommended prophylactic regimens typically involve a single preincisional intravenous dose of a first- or second-generation cephalosporin, although other antibiotic classes may be used based on local epidemiology and formulary structures. In Germany, cefuroxime is the only antibiotic explicitly approved for cesarean prophylaxis, providing reliable coverage of pathogens commonly associated with postcesarean infections [6]. This universal application of PAP represents an essential protective measure but simultaneously introduces a systemic antibiotic exposure during a physiologically dynamic period for postpartum women.

Short-term systemic antibiotic exposure is known to modulate gastrointestinal microbial communities. Even a single perioperative dose can induce compositional and diversity changes in gut microbial communities, affecting metabolic function and the relative abundance of commensal and opportunistic organisms [7,8]. During pregnancy, endocrine and immunometabolic adaptations reshape the gut microbiome toward lower diversity and increased inflammatory tone, resulting in reduced ecological stability. These pregnancy-associated shifts inherently diminish microbial resilience, potentially heightening vulnerability to additional perturbations such as perioperative antibiotics, postoperative dysmotility, or transient inflammatory responses following cesarean delivery [9,10].

Antibiotics administered intravenously during CS reach high systemic levels and can exert direct antimicrobial activity within the intestinal lumen. Pharmacologically active luminal concentrations have been demonstrated for parenterally administered cephalosporins via biliary excretion [11-13]. In addition, postoperative physiological changes, including transient gastrointestinal dysmotility, reduced oral intake, shifts in hormonal and immune activation, analgesic use, and surgical inflammation, further influence microbial composition [14,15]. Clinically, systemic antibiotic exposure is associated with postantibiotic diarrhea, which may involve overgrowth of opportunistic taxa such as *Clostridioides difficile*, illustrating that short, prophylactic antibiotic regimens can perturb microbial homeostasis even in otherwise healthy individuals [16,17]. Together, these mechanisms support the biological plausibility that PAP could induce measurable, short-term compositional changes in the maternal gut microbiome.

Despite this plausibility, empirical evidence specific to postpartum women is remarkably scarce. The maternal gut microbiome during the peripartum and immediate postpartum period has received far less research attention than neonatal colonization or vertical microbial transmission. Alterations in maternal microbial communities could influence metabolic recovery, immune reconstitution, gastrointestinal symptoms, inflammation, wound healing, and overall well-being. However, the postpartum period is underrepresented in microbiome research because traditional, visit-based study designs impose substantial logistical burdens on new mothers. A recent review underscored this gap, highlighting the near absence of high-quality studies evaluating maternal gut microbiome dynamics following CS and PAP [18].

### Rationale for the Study Design

A central methodological consideration is the need for an appropriate comparator. PAP cannot be randomized for ethical reasons as withholding it would contradict the standard of care. Assessing only pre-post changes within CS patients would confound potential antibiotic effects with postoperative physiological responses. A vaginal birth comparison group therefore provides a meaningful counterfactual, capturing physiological postpartum microbial trajectories in the absence of surgical intervention and perioperative antibiotics. However, existing literature is an insufficient substitute for such a comparator, as microbiome datasets vary widely in sequencing region (eg, V1-V2 vs V3-V4), primer selection, read depth, and bioinformatic processing (operational taxonomic unit–based clustering vs amplicon sequence variant [ASV] inference) [19,20]. These methodological discrepancies substantially limit cross-study comparability and hinder valid inference from aggregated external controls. A contemporaneous comparator group is therefore essential for exploratory evaluation of whether postpartum microbial trajectories differ following CS with PAP.

Postpartum research also faces substantial feasibility challenges. New mothers often have limited mobility, competing care demands, and little capacity for additional clinical visits [21]. These barriers disproportionately exclude women from participating in research and limit the representativeness of available data. To address these constraints, we developed a fully remote, participant-centered protocol using at-home stool collection and postal submission. Remote sampling approaches have demonstrated strong feasibility in other areas of microbiome research [22,23], but they have not yet been systematically applied to postpartum maternal populations. Such designs reduce participation burden, enhance accessibility across geographic and socioeconomic contexts, and provide a pragmatic framework for capturing real-world postpartum microbial patterns.

Given the achieved sample size and group imbalance, this study is primarily framed as a feasibility investigation assessing recruitment, adherence, and the performance of remote stool sampling in the early postpartum period. Secondary exploratory analyses aim to characterize maternal gut microbiome composition and diversity following CS with PAP compared with vaginal birth, to evaluate short-term microbial trajectories, and to identify hypothesis-generating taxonomic patterns. These analyses are not powered for confirmatory inference but will inform the design of future, adequately powered studies.

Although PAP is a universal component of CS, its potential impact on the maternal gut microbiome remains insufficiently understood. This protocol addresses this gap by implementing a remote, participant-centered study design that enables feasible, scalable, and ethically grounded investigation of postpartum microbiome dynamics.

### Objectives

The primary objective of the MAMA (Microbiome Changes Due to Antibiotic Prophylaxis in Mothers at Birth) study is to assess the feasibility of a fully remote, nonsite-bound study design for longitudinal gut microbiome research in a peripartum population, including recruitment, sample return, and follow-up completion.

Secondary objectives are to explore compositional changes in the maternal gut microbiome associated with PAP using cefuroxime during CS, compared with vaginal delivery (VD), across the peripartum period.

In this context, the study aims to establish a scalable methodological framework for future remote microbiome studies in maternal health and other vulnerable populations.

## Methods

### Study Design and Setting

**Figure 1.**
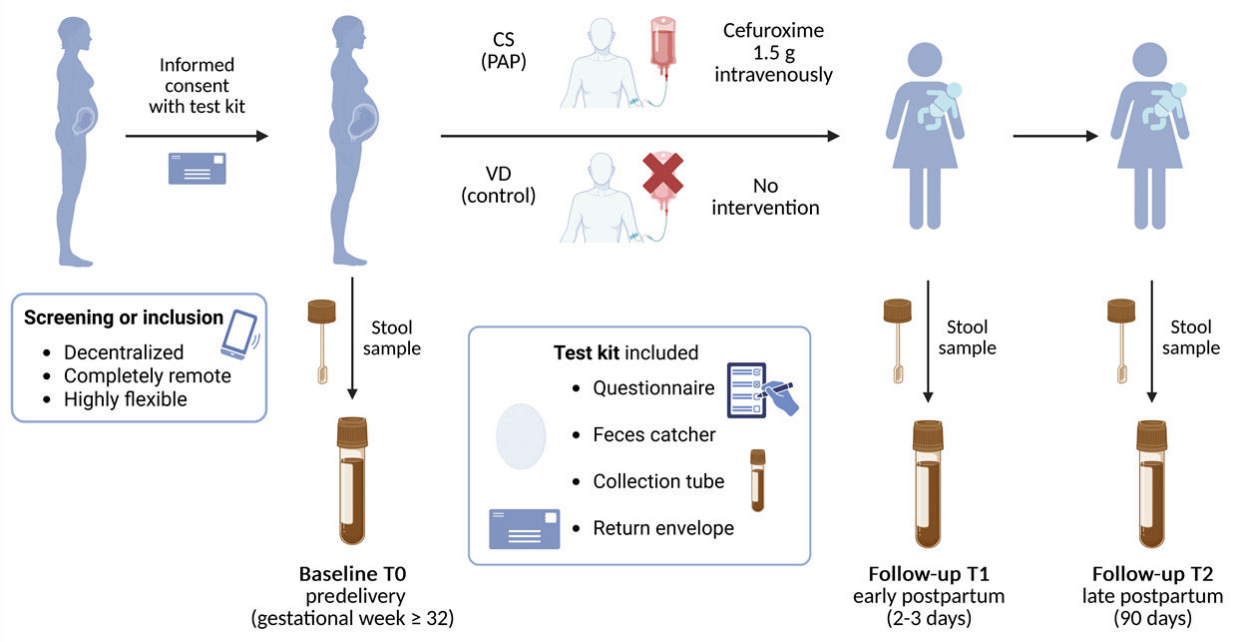
Study design of the MAMA (Microbiome Changes Due to Antibiotic Prophylaxis in Mothers at Birth) trial: fully remote workflow and sample collection schedule. CS: cesarean section; PAP: perioperative antibiotic prophylaxis; VD: vaginal delivery.

The MAMA study was conducted as a prospective, 2-arm observational cohort study comparing women undergoing CS with PAP to women delivering vaginally without intrapartum antibiotic exposure. Recruitment was integrated into routine clinical care, and participants were followed from the third trimester through 90 days postpartum. The study was carried out between May 2022 and March 2024 at 2 German level-1 perinatal centers (Municipal Hospitals of Cologne and St. Marien Hospital Bonn [GFO Kliniken Bonn]) as well as affiliated outpatient providers. An overview of the overall study design and workflow is provided in Figure 1.

The study was originally conceptualized as a hypothesis-testing cohort design aimed at characterizing maternal gut microbiome trajectories in relation to delivery mode and PAP exposure. However, real-world recruitment constraints in this peripartum population resulted in a smaller and unevenly distributed sample size. In view of these conditions, the present protocol primarily serves as a feasibility investigation of a fully decentralized study framework for maternal microbiome research. Accordingly, subsequent microbiome analyses are defined as exploratory and hypothesis-generating rather than confirmatory.

A contemporaneous comparator group was required to interpret microbial patterns observed after CS. Randomization or withholding of the standard of care was ethically impermissible, and pre-post analyses within CS alone would have conflated antibiotic-associated effects with surgical and physiological consequences of operative delivery. VD therefore represented the most appropriate counterfactual, reflecting postpartum microbial trajectories in the absence of PAP and operative stress. External control datasets were not considered suitable due to widely recognized methodological heterogeneity across microbiome studies (eg, primer regions, sequencing workflows, and bioinformatic pipelines), which limits cross-study comparability.

The study was explicitly implemented as a fully remote, decentralized design to minimize participant burden during pregnancy and early postpartum. All study procedures, including screening, informed consent, questionnaire completion, and stool sampling time points, were completed by participants at home. Communication occurred via telephone or institutional email. Preassembled sampling kits were mailed to participants and included written instructions, a stool catcher, a DNA stabilization tube enabling reliable sample quality even when samples remained at home for several days before mailing, a structured paper–based metadata questionnaire, and a preaddressed return envelope. Participants received reminder notifications from the study coordinator regarding upcoming collection time points and missing documentation. The absence of mandatory on-site visits enabled flexible participation, allowing women to complete all study tasks according to their individual schedules and daily responsibilities. This design facilitated nationwide participation and broadened accessibility, particularly for individuals living in rural areas, with limited mobility, or with substantial childcare and occupational commitments.

The study was developed and implemented in close collaboration between microbiologists, clinical pharmacists, obstetricians, and allied health care professionals, ensuring both methodological rigor and practical feasibility. The reporting of this protocol follows the STROBE (Strengthening the Reporting of Observational Studies in Epidemiology) recommendations (Checklist 1) for observational cohort studies, as recommended by the EQUATOR Network [24].

The study was registered in the German Clinical Trials Register (DRKS00027305), which is part of the WHO International Clinical Trials Registry Platform (ICTRP).

Participants were screened and informed by telephone. Upon inclusion, a postal test kit was sent to eligible participants, which included the written informed consent form, a questionnaire, a feces catcher, a stool collection tube, and a preaddressed return envelope. The signed consent form was returned together with the baseline stool sample (T0). Based on the mode of delivery, participants were assigned to either the CS group with PAP (cefuroxime 1.5 g intravenously) or to the control group with VD (no antibiotic exposure). Follow-up samples were collected at 2 to 3 days postpartum (T1) and 90±10 days postpartum (T2). All procedures were conducted by the participants at home, without the need for on-site visits.

### Ethical Considerations

The study was reviewed and approved by the ethics committee of Witten/Herdecke University (274/2021) in February 2022. A substantial amendment covering the addition of a second study center and modifications to the screening process was approved in September 2022. Recruitment commenced after ethical approval, with the first participant enrolled in May 2022, and the last participant in October 2023. Follow-up concluded in March 2024, with the return of the final stool sample. The remote consent procedure was explicitly reviewed and approved by the ethics committee. All participants provided verbal consent during the initial telephone screening and subsequently returned signed written consent forms together with their first stool sample. Study participation was voluntary, and participants could withdraw at any time without providing reasons. No incentives were provided for participation. All data were collected and processed in pseudonymized form in compliance with the General Data Protection Regulation (GDPR). No directly identifying information was accessible to the research team. The data protection concept was developed in collaboration with the institutional data protection officer and reviewed as part of the ethical application. There was no patient or public involvement in the study design or implementation. Pseudonymized raw data will be made available to qualified researchers upon reasonable request to the principal investigator and in accordance with institutional and GDPR requirements. The study was conducted as part of a doctoral project at the Faculty of Health, Witten/Herdecke University.

### Eligibility and Recruitment

Participants were eligible for inclusion if they were aged at least 18 years, in the third trimester of pregnancy, and planned to deliver either by elective CS or VD without medical indication for peripartum antibiotics. Sufficient German language skills were required to complete the informed consent process and written study documentation. Exclusion criteria comprised participation in another clinical study, chronic inflammatory bowel disease (eg, Crohn disease or ulcerative colitis), hematologic or oncologic disease, chronic renal or hepatic disease, and chronic viral infections such as HIV, hepatitis B virus, or hepatitis C virus. Additional exclusions included systemic use of immunosuppressants, virostatics, cytostatics, proton pump inhibitors, or systemic antimicrobial agents (antibiotics or antifungals) within the past 3 months. Women with a foreseeable need for surgery or antimicrobial treatment other than cefuroxime within the study period (90 days postpartum) were also excluded.

Participants were recruited between May 2022 and October 2023 at 2 German level 1 perinatal centers and affiliated outpatient facilities. Eligible women were identified by attending obstetricians and midwives during routine clinical care. To support recruitment beyond the hospital setting and to address recruiting bias, awareness of the study was raised through posters in clinic waiting areas, printed flyers distributed during antenatal visits, and social media announcements via the official channels of the participating institutions. In addition to reaching a broader population, the social media strategy was specifically intended to include women with low-risk pregnancies who received all prenatal care in the outpatient sector and would otherwise have had limited contact with hospital-based recruitment. This approach aimed to reduce recruitment bias toward high-risk pregnancies, such as those with gestational diabetes, multiple gestation, or prior cesarean delivery. Pregnant women and new mothers represent a population with specific logistical and time-related needs, which informed the choice of a decentralized recruitment and study design.

Interested individuals were first approached by clinical staff and subsequently contacted by the study coordinator. Informed consent was obtained remotely in a 2-step process. Following an initial telephone-based eligibility screening and verbal explanation, participants confirmed their consent in writing by mailing back the signed documents together with their first stool sample and completed questionnaire.

Assignment to study groups was based on the actual mode of delivery. Women initially recruited for the control group who ultimately required CS for medical reasons were reallocated to the intervention group. Recruitment concluded after inclusion of 25 women in the CS group and 12 in the vaginal birth group, without reaching the originally planned sample size.

### Data Collection and Sample Handling

Sample collection was performed independently by participants at home as part of the fully remote study workflow. Final group assignment (CS vs VD) was based retrospectively on the actual mode of delivery at birth. Each participant was asked to provide 3 stool samples at predefined time points: during late pregnancy (T0, from gestational week 32 onward), within 2 to 3 days postpartum (T1), and at 90 (±10) days postpartum (T2). These time points were selected to capture baseline microbiome composition, immediate peripartum changes, and long-term recolonization dynamics. For each time point, participants received a sample collection kit that included a collection tube (DNA/RNA Shield Fecal Collection Tube, Zymo Research), a single-use stool catcher (Feces Catcher, Zymo Research), written instructions, and a prepaid return envelope. Paper-based questionnaires accompanied each sample kit and covered maternal health status, medication use, pregnancy and birth-related variables, and lifestyle factors such as nutrition and physical activity. Participants returned both the stool sample and completed questionnaire in the same shipment. Questionnaires were designed to be completed within approximately 10 minutes and spanned 2 to 3 pages, depending on the time point. Incomplete or missing forms were actively followed up by the study coordinator.

Samples were shipped by standard postal service and received by the coordinating study center. Although the exact time between sampling and mailing was not recorded for all participants, no sample exceeded the manufacturer’s recommended stability window.

Upon arrival, samples were documented and immediately stored in a temperature-monitored, alarm-secured refrigerator at 4°C to 8 °C, where they remained until DNA extraction. The median storage duration was 434 days (range: 137‐784 d), with no deviations from the defined storage conditions. Despite the stabilizing effect of DNA/RNA Shield, the wide range of storage periods may still cause subtle bias.

The nucleic acid stabilization buffer (DNA/RNA Shield) ensures preservation of microbial DNA for at least 24 months at ambient temperature (4° C‐25  °C) and indefinite stability when frozen (<−20 °C), according to the manufacturer. Accordingly, all returned samples were accepted for processing. No exclusions were made due to transport delays, damage, or temperature fluctuations.

DNA extraction was performed using the ZymoBIOMICS 96 MagBead DNA Kit (D4308, Zymo Research) on an automated pipetting workstation (epMotion 5075t, Eppendorf). A slightly modified protocol was applied: 200 µL of fecal sample were mixed with 450 µL of lysis buffer and subjected to mechanical disruption using a bead-beating step in a TissueLyzer (30 Hz; 5 min). DNA was eluted in 50 µL of nuclease-free water and stored at 4 °C to 8  °C until library preparation. For amplification of the V3-V4 region of the 16S rRNA gene, the Quick-16S Next Generation Sequencing Library Prep Kit (D6400, Zymo Research) was used with 341f/806r primers. The protocol was modified by using half reaction volumes and performing 30 polymerase chain reaction (PCR) cycles. No fluorometric quantification was performed prior to amplification; however, postamplification quality control confirmed consistent DNA concentrations across all samples, and no samples had to be excluded due to insufficient yield or quality. Sequencing was conducted on an Illumina MiSeq platform using the MiSeq Reagent Kit v3 (600-cycle, paired-end 301 bp; MS-102‐3003, Illumina). Cluster generation occurred onboard the MiSeq instrument. Pooled libraries were denatured and diluted according to the manufacturer’s protocol and loaded at a final concentration of 12 pM. A PhiX control library (15%) was spiked in to increase run diversity and monitor sequencing quality. Quality controls included 2 extraction blanks consisting of lysis buffer from the DNA extraction kit, one of which was carried through library preparation, and 1 PCR-negative control (nuclease-free water). No spike-in standards for absolute quantification were used. A positive control from the Quick-16S Next Generation Sequencing Library Prep Kit (ZymoBIOMICS Microbial Community DNA Standard) was processed alongside study samples. Mock community controls beyond this kit-derived standard were not included. All study samples and controls were processed and sequenced in a single batch to avoid inter-batch variation.

### Microbiome Analysis and Bioinformatics Pipeline

Raw sequencing data will be processed in a QIIME2 (Quantitative Insights Into Microbial Ecology 2; version 2023.2; QIIME 2 Development Team) environment [25], executed within the Qiita platform (Knight Lab, University of California San Diego) [26]. Upon import into Qiita, FASTQ files will undergo automated metadata validation and quality filtering, including removal of low-quality reads, trimming of low-quality ends according to run-specific quality profiles. Adapter and primer sequences will be removed using cutadapt (version 4.2; Max Planck Institute for Developmental Biology) prior to downstream denoising. ASVs will be inferred using Deblur (Knight Lab, University of California San Diego) [27]. Deblur performs error correction and dereplication to generate high-resolution ASVs, while removing putative chimeras and low-abundance artefactual sequences as part of its standard denoising workflow. Because 16S amplicon sequencing provides only relative abundance estimates, absolute bacterial load is not quantified. This will be considered as a possible source of bias when interpreting results. Taxonomic assignment will be conducted using the pretrained Greengenes2 2024.9 full-length classifier, based on the corresponding Greengenes2 2024.9 reference taxonomy [28]. Sequences classified as chloroplast or mitochondria will be identified using Greengenes (version 13.8) and excluded [29]. Representative sequences will be generated within Qiita, and phylogenetic placement will rely on the Greengenes (version 13.8) 99% identity reference tree. Extraction blanks and PCR negative controls will be processed alongside study samples to detect potential laboratory or reagent contamination. Mock community controls were not included. Samples will be rarefied to a minimum depth of 30,000 reads for alpha diversity and beta diversity analyses. Samples below this threshold will be excluded from diversity metrics but may be retained for descriptive taxonomic summaries.

Analyses will follow a 2-level structure. First, community-level analyses where ASV-based distance matrices (Bray-Curtis, and weighted and unweighted UniFrac) will be used to assess group differences via permutational multivariate ANOVA [30]. Longitudinal microbiome changes will be evaluated by comparing within-individual distance differences across time intervals using Mann-Whitney *U* tests, visualized via principal coordinates analysis. Second, taxonomic-level analyses, in which higher-rank taxonomic profiles (phylum, genus, and species, where available) are summarized descriptively. Hypothesis testing at the taxonomic level will only be conducted if community-level differences emerge, to avoid overinterpretation of sparse compositional data. Given the modest cohort size, all analyses are designed for hypothesis-generating, exploratory interpretation.

Alpha diversity (Shannon index, observed features, and Faith phylogenetic diversity) and beta diversity (Bray-Curtis, and weighted and unweighted UniFrac) will be calculated to describe within- and between-sample diversity. Group comparisons will be analyzed using the Wilcoxon rank-sum or Kruskal-Wallis tests for alpha diversity and permutational multivariate ANOVA for beta diversity. Differential-abundance analysis will be performed using the Analysis of Composition of Microbiomes II (ANCOM-II) [31], a method developed for sparse compositional microbiome data. Given the modest sample size, differential-abundance findings will be interpreted exploratorily. All downstream analyses and visualizations will be performed in QIIME2 and a Python-based environment (Python Software Foundation). Workflow steps, parameters, and software versions will be version-controlled to ensure reproducibility. In addition to taxonomic data, analyses will integrate metadata on parity, medication use, and dietary habits. Associations between microbiome composition and clinical, lifestyle, or sociodemographic variables will be explored through correlation analyses and, where applicable, multivariate approaches such as redundancy analysis or generalized linear models.

Although DNA/RNA Shield stabilizes microbial nucleic acids, prolonged refrigerated storage before extraction may introduce subtle compositional shifts. This potential source of bias will be considered during interpretation.

### Outcomes

The primary outcomes of this protocol are feasibility indicators that assess the performance of the fully remote study design. These include the proportion of contacted women who enrolled; sample and questionnaire return rates at T0, T1, and T2; timeliness of sample returns relative to the predefined sampling windows; and overall participant retention across study time points. Together, these measures evaluate compliance, protocol adherence, and the suitability and scalability of the decentralized workflow for maternal microbiome research.

Microbiome-related outcomes are exploratory and focus on compositional rather than absolute changes in microbial communities, as 16S rRNA-sequencing yields relative abundances. These outcomes include alpha diversity metrics (eg, Shannon index and Chao1 richness), beta diversity metrics (eg, Bray-Curtis dissimilarity, and weighted and unweighted UniFrac distances), and relative abundances of bacterial taxa across taxonomic levels from phylum to genus with ASV-level feature tables used for technical analyses. Exploratory identification of marker taxa may be performed where feasible. No diagnostic or clinical interpretations of “dysbiosis” will be drawn.

In line with the observational design and modest, imbalanced sample size, all hypotheses are nonconfirmatory and serve to guide hypothesis-generating analyses. We hypothesize that women undergoing CS with PAP will exhibit a transient reduction in alpha diversity at T1 compared to VD; that compositional perturbations at T1 will at least partially resolve by T2; and that the relative abundance of *Clostridioides difficile* will be higher at T1 in the CS group than in VD. These hypotheses reflect biologically plausible patterns without implying confirmatory testing.

Exploratory analyses will consider associations between microbiome features and maternal metadata such as parity, medication intake, and dietary patterns, where feasible. Subgroup analyses may be limited by sample size constraints, and residual confounding is expected.

Outcomes will be described both cross-sectionally (CS vs VD at each time point) and longitudinally (across T0→T1→T2 within individuals as well as within groups). The statistical procedures for these analyses are outlined in the subsequent section.

### Statistical Analysis Plan

The following procedures specify how the predefined outcomes will be analyzed. The analysis will follow an exploratory framework. Although simple, biologically plausible hypotheses have been specified, no formal power calculation was performed. Consequently, results will be interpreted as hypothesis-generating rather than confirmatory.

Feasibility outcomes will be summarized descriptively using proportions, medians, and ranges. Timing of sample returns relative to the predefined collection windows (T0, T1, and T2) will be evaluated by calculating time intervals (in d) between the target and actual return dates. These intervals will be summarized using medians and IQR and compared between delivery groups where appropriate. Reasons for loss to follow-up (eg, no sample returned) will be documented, and analyses will be performed on available cases without imputation of missing data. This analysis will help assess protocol adherence and identify potential logistical barriers in the home-based study model.

To address the previously defined microbiome outcomes, the following statistical approaches will be applied: group comparisons between CS and VD will be performed for each time point (T0, T1, and T2) and longitudinally within participants as well as within groups, using all available paired samples (eg, T0-T1 or T1-T2), where applicable. Alpha diversity metrics (eg, Shannon index and Chao1 richness) will be compared using nonparametric tests (Wilcoxon rank-sum or Kruskal-Wallis test). Beta diversity will be evaluated using permutational multivariate ANOVA based on distance matrices (eg, Bray-Curtis and UniFrac). Visualization will include principal coordinates analysis.

Analyses will follow a 2-level structure, with primary community-level testing and secondary, exploratory taxonomic-level summaries.

Relative abundances of microbial taxa at different taxonomic levels will be summarized descriptively.

Exploratory differential-abundance analyses will be performed using ANCOM-II, a method suitable for sparse compositional data that incorporates its own procedures for controlling false discoveries. Taxonomic-level hypothesis testing will only be performed if community-level analyses indicate meaningful group differences.

Missing data will not be imputed. Samples with insufficient sequencing depth will be excluded from diversity analyses but may be retained for descriptive taxonomic summaries. Sensitivity analyses will be considered to evaluate the robustness of key findings. Sensitivity analyses may include rerunning key comparisons with alternative distance metrics or differential-abundance methods. If imbalances in relevant maternal variables (eg, prevalence of gestational diabetes) are observed between study groups, multivariate statistical models (eg, generalized linear models and redundancy analysis) will be applied to adjust for potential confounding effects. Stratified analyses or subgroup comparisons may also be conducted if sample size permits.

All statistical analyses will be performed in Python, using QIIME 2-compatible plugins (eg, ANCOM-II) and standard scientific Python libraries. Software versions and analysis scripts will be documented for reproducibility.

### Dissemination Plan

The findings of this study will be disseminated through open-access, peer-reviewed publications and presentations at clinical and scientific meetings. This protocol represents the first of 2 planned manuscripts. The main results paper is anticipated for submission in the first half of 2026. Upon publication of the results, raw sequencing data (FASTQ files) will be deposited in the European Nucleotide Archive. The bioinformatic workflow (Python/QIIME 2 scripts) will be made available in a version-controlled repository or provided upon reasonable request to support transparency and reproducibility. Pseudonymized metadata will be shared with qualified researchers upon request in compliance with GDPR and institutional requirements.

Interested participants may request a plain-language summary of the study results in German. Study registration in DRKS/WHO ICTRP ensures continuous transparency, and future updates to the study outputs will be linked to the registry entry. The decentralized design and methodological framework developed here are intended to inform larger maternal health studies and may be adapted for broader applications in remote microbiome research.

## Results

**Figure 2.**
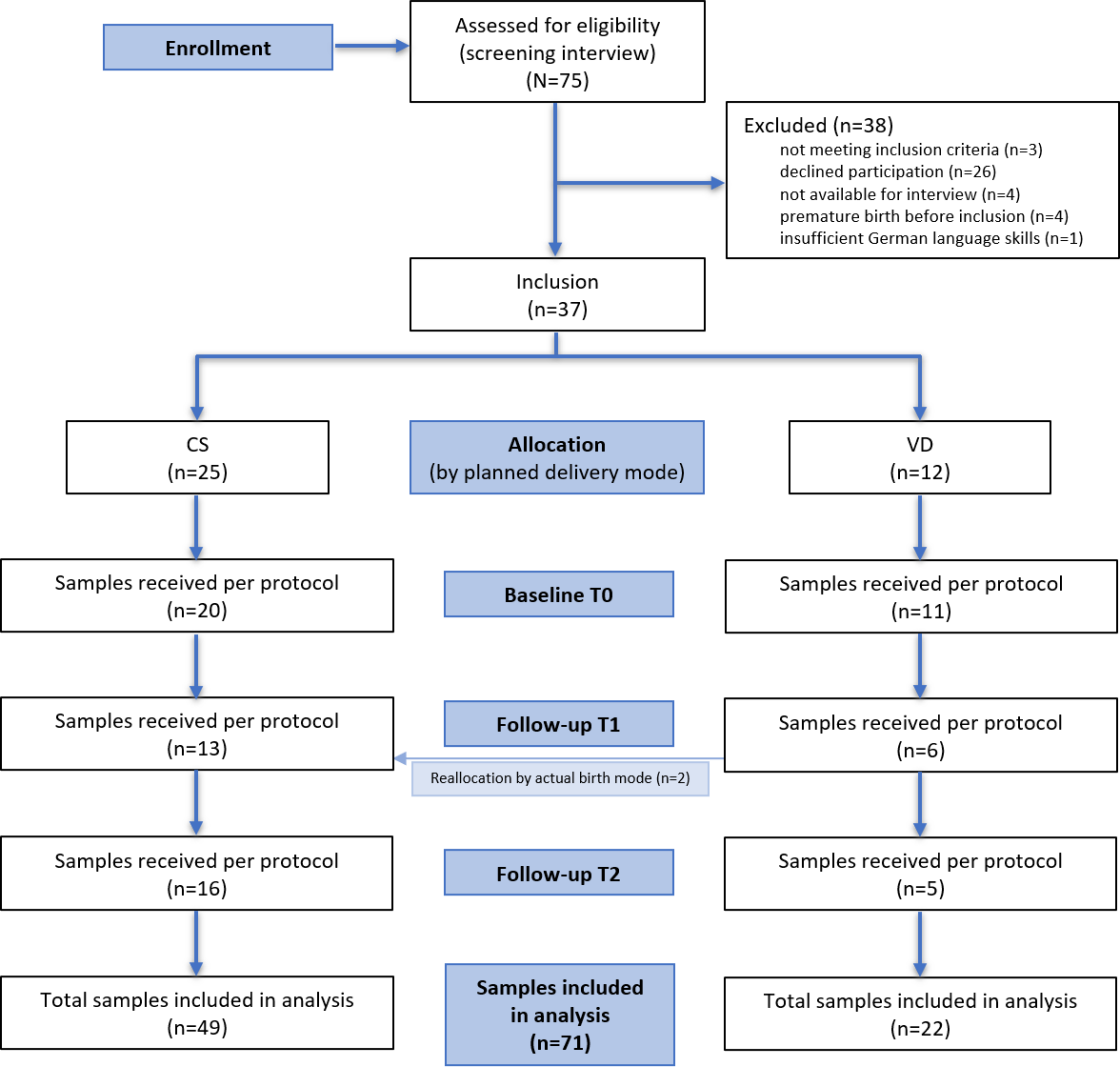
Flowchart of participant enrollment, allocation, follow-up, and analysis in the MAMA (Microbiome Changes Due to Antibiotic Prophylaxis in Mothers at Birth) study, in line with the STROBE (Strengthening the Reporting of Observational Studies in Epidemiology) recommendations for observational studies. CS: cesarean section; VD: vaginal delivery.

The recruitment and sample collection phase of the study has been completed. Between May 2022 and October 2023, a total of 37 participants were enrolled, including 25 women in the CS group and 12 in the VD group. Recruitment concluded below the originally planned sample size, reflecting real-world constraints in this peripartum population. Follow-up was completed with receipt of the final stool sample in March 2024. An overview of baseline characteristics of participants who returned at least one stool sample is presented in Table 1. DNA extraction and 16S rRNA gene sequencing were completed in a single batch in October 2024. All samples passed quality control and were retained for downstream analyses. An overview of included and excluded samples by group and time point is provided in Figure 2. Bioinformatic processing and statistical analyses were initiated in June 2025 and were ongoing as of December 2025.

**Table 1.** Baseline characteristics of participants who submitted at least 1 sample (N=31)^a^.

Characteristic		Total (N=31)	CS^b^ with PAP^c^ (n=20)		VD^d^ without PAP (n=11)	
Continuous variables						
Age (y), median (IQR)		37 (33-39)	37 (33-39)		36 (33-38)	
Categorical variables, n (**%**)						
Delivery mode						
CS		20 (65)	20 (100)		—^e^	
VD		11 (35)	—		11 (100)	
Multiple births						
Yes		2 (6)	2 (10)		0 (0)	
No		29 (94)	18 (90)		11 (100)	
Parity						
0		12 (39)	9 (45)		3 (27)	
1		13 (42)	5 (25)		8 (73)	
2		5 (16)	5 (25)		0 (0)	
3		0 (0)	0 (0)		0 (0)	
4		0 (0)	0 (0)		0 (0)	
5		1 (3)	1 (5)		0 (0)	
>1		19 (61)	11 (55)		8 (73)	

a Only participants in the CS group received PAP; no antibiotics were administered in the VD group.

b CS: cesarean section.

c PAP: perioperative antibiotic prophylaxis.

d VD: vaginal delivery.

e Not applicable.

A per-protocol sample was defined as a stool sample received within the prespecified time window and passing quality control. Protocol deviation was defined as exclusion from the per protocol set due to intake of antimicrobial agents (antibiotic or antifungal) other than the study medication or as sample collection outside the defined protocol windows and could not be assigned to a valid time point. Two women, who were initially allocated to the vaginal delivery group, delivered by CS and were therefore analyzed in the CS group; these women are counted under vaginal delivery at baseline (T0) and under CS at follow-up (T1 and T2). Numbers reported per group reflect participants analyzed at each time point; totals across groups were: T0 (n=31), T1 (n=19), T2 (n=21), and final analysis (N=71).

## Discussion

### Principal Findings

This study evaluates the feasibility of a fully decentralized study model in a peripartum population, addressing practical barriers to participation in longitudinal microbiome research. Within this feasible framework, we anticipate exploratory, compositional alterations of the maternal gut microbiome following CS with PAP, most pronounced shortly after birth and attenuating over time. Given the limited and imbalanced sample size, these microbiome analyses are considered hypothesis-generating rather than confirmatory. In this context, the MAMA study exemplifies how to address practical barriers frequently encountered in peripartum research, including logistical challenges, limited time resources, and competing family responsibilities, which have made participation in longitudinal microbiome studies particularly difficult for pregnant women and new mothers.

We addressed these limitations by implementing a fully off-site study model that enables microbiome research during pregnancy and early motherhood—without requiring any in-person study visits. This approach not only facilitates participation across geographic and social boundaries but also integrates scientific inquiry into the realities of maternal life. The fully remote design, with telephone-based recruitment and postal sample return, was logistically feasible and well-accepted by participants. Particularly for pregnant women with limited mobility, young children, or those living far from clinical centers, the ability to participate from home appeared to have lowered the barrier to enrollment. Moreover, the participant-based approach allowed for maximum flexibility in scheduling the informed consent process. Participants were able to choose individually convenient times, including evenings and weekends, and rescheduling was possible at any time. In light of the exploratory nature and limited sample size, the primary contribution of this protocol lies in evaluating the feasibility of a fully remote study model in a peripartum population, while microbiome-related analyses are planned as secondary, exploratory outcomes.

Although digital data collection might be considered more efficient, a fully digital questionnaire system was not implemented due to institutional constraints and concerns regarding digital accessibility. Instead, paper-based questionnaires were included in each sample kit, aligning with the postal return of stool samples. This low-threshold approach ensured that participants could complete the forms directly when packaging their sample, without requiring technical equipment or online access. To maximize usability, all materials were written in plain language, following the principle of one idea per sentence, with no more than 15 words per sentence. Where possible, checkbox-style response options were used to reduce cognitive load and completion time.

Beyond logistical feasibility, participant acceptance also emerged as a relevant aspect of the remote study design, as several participants actively expressed interest in the study’s scientific goals and asked to be informed of the results. This feedback underscores the importance of building research models that are not only scientifically robust but also accessible, respectful of participants’ realities, and responsive to their curiosity and health concerns.

Despite growing awareness of the microbiome’s role in health, none of the participating women reported using probiotic supplements during the study period. This observation highlights a potential disconnect between perceived relevance and actionable guidance. The present study may provide a foundation for future research exploring the efficacy and safety of probiotic or dietary interventions during pregnancy and post partum.

At the same time, the implementation of a fully non–site-bound model presented specific challenges. In the VD group, it was difficult to anticipate the exact timing of delivery and thus ensure timely sample collection at T0 and T1. Participants were encouraged to prepare the sampling kit in advance and bring it to the hospital in their birth bag, but adherence to this instruction was not always possible. Additionally, logistical issues were encountered early in the study due to the use of time-limited digital postage codes. As these codes expired before participants mailed their samples, significant mailing delays occurred. The issue was resolved by switching to traditional postage stamps, which ensured reliable sample dispatch without further complications. Although this did not result in sample loss, it created confusion and required additional follow-up communication. These experiences highlight the need for practical guidance and reliable infrastructure when conducting off-site clinical research.

Recruitment was also affected by varying levels of institutional engagement across study sites. While the home-based design reduced participant burden, it still required initial support from clinical staff to initiate contact or distribute study materials. Staff at one study center demonstrated strong interdisciplinary cooperation, with committed medical professionals actively facilitating participant outreach. This differed significantly in the other study center, where limited personnel resources and skepticism about participants’ willingness to provide samples postpartum led to reluctance in supporting recruitment. This ultimately contributed to the premature closure of enrollment before the initially planned sample size was reached. This limitation in institutional support directly impacted the final sample size, which remained below target and unevenly distributed between the 2 study arms. In addition, several participants had to be excluded during the study due to protocol violations, such as postpartum antibiotic use identified retrospectively through questionnaire data, or delayed sample return beyond the predefined protocol windows. Despite these constraints, the sampling scheme captured three critical time points—before delivery, shortly after birth, and during postpartum recovery—enabling the identification of both acute and long-term changes in microbial composition.

While the observational design does not aim to establish causal effects, the resulting dataset is expected to yield meaningful insights into temporal microbiome dynamics in the peripartum period. Due to the exploratory nature of this observational study, no formal measures to control for bias resulting from group imbalance or participant dropouts were applied at the design stage. Potential confounding will be addressed during statistical analysis through stratification and adjustment, as appropriate. To this end, several relevant variables were prospectively recorded, including parity, medication or supplement use, diet, and allergies. Where numbers allow, these factors will be considered in stratified or adjusted analyses; however, residual confounding is likely given the modest and imbalanced sample size. Limitations in group comparability will be transparently addressed in the planned results publication in 2026.

### Limitations

Importantly, the modest and uneven sample sizes between study groups substantially limit statistical power and preclude confirmatory inference. As microbiome analyses are based on relative abundances derived from 16S rRNA sequencing, absolute bacterial load was not quantified; consequently, the lack of spike-in controls or quantitative PCR may affect the interpretation of apparent taxonomic enrichments or depletions. Consequently, microbiome-related analyses must be interpreted with caution, as they are susceptible to random variation and cannot reliably detect small or moderate effect sizes. This limitation directly informed the prioritization of feasibility outcomes as primary end points, while microbiome outcomes are framed as secondary and hypothesis-generating.

In addition, the unbalanced group sizes and incomplete longitudinal follow-up further restrict generalizability and limit the ability to perform robust subgroup or multivariable analyses. Consequently, the present study is not intended to provide definitive estimates of effect magnitude, but rather to delineate plausible patterns and inform the design of future, adequately powered investigations.

### Comparison With Prior Work

While several longitudinal studies have investigated peripartum changes in the maternal gut microbiome [9,32], existing evidence does not address the specific impact of CS and, in particular, PAP nor their potential association with dysbiosis in the postpartum period. This represents a relevant gap in current research [18], given the widespread use of antibiotic prophylaxis during CS. Microbiome dysbiosis is commonly characterized by compositional shifts, including reduced diversity and altered relative abundances of immunologically and metabolically relevant taxa. Such patterns have been associated with adverse pregnancy outcomes, for example, in preeclampsia, underscoring the potential relevance of maternal gut microbiome alterations during and after pregnancy [33].

Perturbations of the maternal gut microbiome induced by CS with PAP may have implications that extend beyond the immediate postpartum period. Pregnancy is characterized by profound metabolic and immunological adaptations that facilitate fetal tolerance while supporting increased maternal energy demands, processes that are closely intertwined with gut microbial composition [10]. While these adaptations are physiologically beneficial during pregnancy, alterations of the maternal microbiome in the postpartum period may be relevant for subsequent pregnancies. Evidence from a longitudinal cohort study suggests that specific gut microbiome patterns can precede the clinical diagnosis of gestational diabetes, indicating a potential link between microbial composition and later metabolic dysregulation [34]. In addition, dysbiosis in other maternal microbial niches, such as the vaginal microbiome, has been associated with pregnancy-related complications, including gestational diabetes, preterm birth, preeclampsia, and miscarriage [35]. Although these associations are exploratory and do not imply causality, they underscore the importance of longitudinal maternal microbiome research that extends beyond a single pregnancy.

Future studies may build on this protocol by recruiting larger and more balanced cohorts to enable adequately powered analyses. Incorporating absolute quantification approaches, such as quantitative PCR or spike-in standards, and extending longitudinal follow-up across multiple pregnancies would allow more robust assessment of maternal microbiome dynamics and their potential clinical relevance. The remote, participant-centered design evaluated here provides a feasible framework for such future larger-scale investigations.

### Conclusions

The MAMA study demonstrates the feasibility of conducting fully decentralized microbiome research in a peripartum population without requiring on-site study visits. By integrating recruitment, informed consent, and longitudinal sample collection into participants’ everyday lives through flexible, patient-oriented logistics, this protocol provides a practical and acceptable framework for maternal microbiome research during pregnancy and early post partum.

Given the exploratory design, modest sample size, and group imbalance, this study is not intended to support confirmatory conclusions regarding microbiome alterations following CS with perioperative antibiotic prophylaxis. Accordingly, microbiome-related outcomes should be interpreted as hypothesis-generating, while the primary contribution of this work lies in evaluating and documenting the feasibility of a remote study model in a clinically and logistically sensitive population.

By deliberately centering the maternal perspective and prioritizing maternal health, the MAMA study addresses an underrepresented aspect of perinatal research and illustrates that methodological rigor can be achieved alongside high accessibility for pregnant women and new mothers through an interdisciplinary approach. This framework enables future, larger-scale investigations aimed at robustly characterizing maternal microbiome dynamics and their potential relevance across and beyond pregnancy.

## Supplementary material

10.2196/84909 Checklist 1 Revised STROBE checklist for the MAMA study protocol on perioperative antibiotic prophylaxis in cesarean section.

## Abbreviations

ANCOM-II: Analysis of Composition of Microbiomes II
ASV: amplicon sequence variant
CS: cesarean section
DRKS: German Clinical Trials Register (Deutsches Register Klinischer Studien)
GDPR: General Data Protection Regulation
ICTRP: International Clinical Trials Registry Platform
MAMA: Microbiome Changes Due to Antibiotic Prophylaxis in Mothers at Birth
PAP: perioperative antibiotic prophylaxis
PCR: polymerase chain reaction
QIIME 2: Quantitative Insights Into Microbial Ecology 2
STROBE: Strengthening the Reporting of Observational Studies in Epidemiology
VD: vaginal delivery
WHO: World Health Organization
