# Supplementary material for: Perioperative Antibiotic Prophylaxis in Cesarean Section and the Maternal Gut Microbiome: Protocol for a Remote Observational Cohort Study
Source: JMIR Res Protoc. 2026 Apr 22;15:e84909. doi: 10.2196/84909 (PMC13102285; doi:10.2196/84909)
Supplement: Checklist 1 [file resprot-v15-e84909-s001.pdf]

STROBE Statement—checklist of items that should be included in reports of observational studies
"Perioperative Antibiotic Prophylaxis in Cesarean Section and the Maternal Gut Microbiome: Protocol for a Remote Observational Cohort Study"
(Feles et Mattner)

	Item No.	Recommendation	Page No.	Relevant text from manuscript
**Title and abstract**	1	(*a*) Indicate the study's design with a commonly used term in the title or the abstract	1	*Perioperative Antibiotic Prophylaxis in Cesarean Section and the Maternal Gut Microbiome: Protocol for a Remote Observational Cohort Study* (Title)<br>*"The […] MAMA study is a prospective, two-arm observational cohort study"* (p. 1)
		(*b*) Provide in the abstract an informative and balanced summary of what was done and what was found	2	*"Recruitment occurred between May 2022 and October 2023, with 37 women enrolled (25 CS with PAP; 12 VD). Follow-up was completed with receipt of the final stool sample in March 2024. DNA extraction and sequencing were completed in a single batch in October 2024. Bioinformatic processing and statistical analyses were initiated in June 2025 and are ongoing as of December 2025. Results from the exploratory microbiome analyses are*

				expected to be published in 2026." "*This protocol demonstrates the feasibility of conducting fully decentralized, longitudinal microbiome research in a peripartum population without requiring on-site visits.*" (p. 2)
**Introduction**				
Background/rationale	2	Explain the scientific background and rationale for the investigation being reported	3	*"Short-term systemic antibiotic exposure is known to modulate gastrointestinal microbial communities. […] During pregnancy, endocrine and immunometabolic adaptations reshape the gut microbiome toward lower diversity and increased inflammatory tone, resulting in reduced ecological stability. Despite this plausibility, empirical evidence specific to postpartum women is remarkably scarce."* (p. 3)
Objectives	3	State specific objectives, including any prespecified hypotheses	12	*"The primary objective of the MAMA study is to assess the feasibility of a fully remote, non-site-bound study design for longitudinal gut microbiome research in a peripartum population, including*

				recruitment, sample return, and follow-up completion. Secondary objectives are to explore compositional changes in the maternal gut microbiome associated with PAP using cefuroxime during CS." (p. 5) *"We hypothesize that women undergoing CS with PAP will exhibit a transient reduction in alpha-diversity at T1 compared to VD; that compositional perturbations at T1 will at least partially resolve by T2; and that the relative abundance of Clostridioides difficile will be higher at T1 in the CS group than in VD."* (p. 12)
**Methods**				
Study design	4	Present key elements of study design early in the paper	5; 9	*"conducted as a prospective, two-arm observational cohort study comparing women undergoing CS with PAP to women delivering vaginally without intrapartum antibiotic exposure"* (p. 5) "*Each participant was asked to provide three stool samples at predefined time points: during late pregnancy (T0, from gestational week 32 onwards),*

				within 2-3 days postpartum (T1), and at 90 (±10) days postpartum (T2).” (p. 9)
Setting	5	Describe the setting, locations, and relevant dates, including periods of recruitment, exposure, follow-up, and data collection	6	*“Recruitment commenced after ethics approval, with the first participant enrolled in May 2022, and the last participant in October 2023. Follow-up concluded in March 2024, with the return of the final stool sample.”* (p. 7)<br>*“All study procedures, including screening, informed consent, questionnaire completion, and stool sampling time points, were completed by participants at home.”* (p. 6)
Participants	6	(*a*) *Cohort study*—Give the eligibility criteria, and the sources and methods of selection of participants. Describe methods of follow-up<br>~~*Case-control study*—Give the eligibility criteria, and the sources and methods of case ascertainment and control selection. Give the rationale for the choice of cases and controls~~<br>~~*Cross-sectional study*—Give the eligibility criteria, and the sources and methods of selection of participants~~	8;<br>Figure 1 (p. 7)	
		~~(*b*) *Cohort study*—For matched studies, give matching criteria and number of exposed and unexposed~~<br>~~*Case-control study*—For matched studies, give matching criteria and the number of controls per case~~	not applicable	
Variables	7	Clearly define all outcomes, exposures, predictors, potential confounders, and effect modifiers. Give diagnostic criteria, if applicable	12	
Data sources/ measurement	8*	For each variable of interest, give sources of data and details of methods of assessment (measurement). Describe comparability of assessment methods if there is more than one group	12-14	*“Feasibility outcomes will be summarized descriptively using*

				proportions, medians, and ranges." (p. 13)<br>"Group comparisons between CS and VD will be performed for each time point (T0, T1, T2) and longitudinally within participants as well as within groups, using all available paired samples (e.g., T0-T1 or T1-T2), where applicable. […]Analyses will follow a two-level structure, with primary community-level testing and secondary, exploratory taxonomic-level summaries." (p. 13)
Bias	9	Describe any efforts to address potential sources of bias	8; 11; 12; 18-19	"[…] *the social media strategy was specifically intended to include women with low-risk pregnancies who received all prenatal care in the outpatient sector and would otherwise have had limited contact with hospital-based recruitment. This approach aimed to reduce recruitment bias toward high-risk pregnancies."* (p. 8)<br>"*Because 16S amplicon sequencing provides only relative abundance estimates, absolute bacterial load is not quantified. This will be*

				considered as a possible source of bias when interpreting results." (p. 11)<br>"*Although DNA/RNA Shield stabilizes microbial nucleic acids, prolonged refrigerated storage before extraction may introduce subtle compositional shifts. This potential source of bias will be considered during interpretation."* (p. 12)<br>"*Due to the exploratory nature of this observational study, no formal measures to control for bias resulting from group imbalance or participant dropouts were applied at the design stage. Potential confounding will be addressed during statistical analysis through stratification and adjustment, as appropriate."* (p. 18-19)
Study size	10	Explain how the study size was arrived at	9	*"Recruitment concluded after inclusion of 25 women in the CS group and 12 in the vaginal birth group, without reaching the originally planned sample size."* (p. 9)

Continued on next page

Quantitative variables	11	Explain how quantitative variables were handled in the analyses. If applicable, describe which groupings were chosen and why	22	*"This manuscript describes a study protocol only. No study data were generated or analyzed at this stage."* (p. 22)
Statistical methods	12	(*a*) Describe all statistical methods, including those used to control for confounding	12-14	
		(*b*) Describe any methods used to examine subgroups and interactions	14; 19	*"Stratified analyses or subgroup comparisons may also be conducted if sample size permits."* (p. 14) "*Limitations in group comparability will be transparently addressed in the planned results publication in 2026*." (p. 19)
		(*c*) Explain how missing data were addressed	13	*"Missing data will not be imputed."* (p. 13)
		(*d*) *Cohort study*—If applicable, explain how loss to follow-up was addressed<br>~~*Case-control study*—If applicable, explain how matching of cases and controls was addressed~~<br>~~*Cross-sectional study*—If applicable, describe analytical methods taking account of sampling strategy~~	13	*"Reasons for loss to follow-up (e.g., no sample returned) will be documented, and analyses will be performed on available cases without imputation of missing data."* (p. 13)
		(*e*) Describe any sensitivity analyses	not applicable	*Note: Not applicable in this protocol; sensitivity analyses will be considered in the results publication.*
**Results**				
Participants	13*	(a) Report numbers of individuals at each stage of study—eg numbers potentially eligible, examined for eligibility, confirmed eligible, included in the study, completing follow-up, and analysed	16	Figure 2
		(b) Give reasons for non-participation at each stage	16	Figure 2
		(c) Consider use of a flow diagram	16	Figure 2
Descriptive data	14*	(a) Give characteristics of study participants (eg demographic, clinical, social) and information on exposures and potential confounders	15	Table 1

				Note: Additional participant characteristics, exposures, and potential confounders will be reported in the subsequent results publication.
		(b) Indicate number of participants with missing data for each variable of interest	Table 1 (p. 15)	*Note: No baseline variables had missing data; missing outcome data will be reported in the subsequent results publication.*
		(c) *Cohort study*—Summarise follow-up time (eg, average and total amount)	13	*"Timing of sample returns relative to the predefined collection windows (T0, T1, T2) will be evaluated by calculating time intervals (in days) between the target and actual return dates."* (p. 13)
Outcome data	15*	*Cohort study*—Report numbers of outcome events or summary measures over time	not applicable	*Note: Not applicable in this protocol; numbers of outcome will be reported in the results publication.*
		~~*Case-control study*—Report numbers in each exposure category, or summary measures of exposure~~		
		~~*Cross-sectional study*—Report numbers of outcome events or summary measures~~		
Main results	16	(*a*) Give unadjusted estimates and, if applicable, confounder-adjusted estimates and their precision (eg, 95% confidence interval). Make clear which confounders were adjusted for and why they were included	not applicable	*Note: Not applicable in this protocol; precision analyses will be considered in the results publication.*
		(*b*) Report category boundaries when continuous variables were categorized	not applicable	*Note: Not applicable in this protocol; continuous variables will be published in the results publication.*

		(*c*) If relevant, consider translating estimates of relative risk into absolute risk for a meaningful time period	not applicable	

Continued on next page

Other analyses	17	Report other analyses done—eg analyses of subgroups and interactions, and sensitivity analyses	19	*"Potential confounding will be addressed during statistical analysis through stratification and adjustment, as appropriate. To this end, several relevant variables were prospectively recorded, including parity, medication or supplement use, diet and allergies. Where numbers allow, these factors will be considered in stratified or adjusted analyses; however, residual confounding is likely given the modest and imbalanced sample size. Limitations in group comparability will be transparently addressed in the planned results publication in 2026." (p. 19)*
**Discussion**				
Key results	18	Summarise key results with reference to study objectives	20	*"The MAMA study demonstrates the feasibility of conducting fully decentralized microbiome research in a peripartum population without requiring on-site study visits. […] This framework enables future, larger-scale investigations aimed at robustly characterizing maternal microbiome dynamics."* (p. 20)
Limitations	19	Discuss limitations of the study, taking into account sources of potential bias or imprecision. Discuss both direction and magnitude of any potential bias	12; 17	*"Subgroup analyses may be limited by sample size constraints, and residual confounding is expected."* (p. 12)

				“*Given the limited and imbalanced sample size, these microbiome analyses are considered hypothesis-generating rather than confirmatory.”* (p. 17)
Interpretation	20	Give a cautious overall interpretation of results considering objectives, limitations, multiplicity of analyses, results from similar studies, and other relevant evidence	20	*“This protocol provides a practical and acceptable framework for maternal microbiome research during pregnancy and early postpartum. […]Accordingly, microbiome-related outcomes should be interpreted as hypothesis-generating, while the primary contribution of this work lies in evaluating and documenting the feasibility of a remote study model in a clinically and logistically sensitive population.”* (p. 20)
Generalisability	21	Discuss the generalisability (external validity) of the study results	20	*“[The MAMA study] illustrates that methodological rigor can be achieved alongside high accessibility. […] This framework enables future, larger-scale investigations aimed at robustly characterizing maternal microbiome dynamics and their potential relevance across and beyond pregnancy.”* (p. 20)
**Other information**				
Funding	22	Give the source of funding and the role of the funders for the present study and, if applicable, for the original study on which the present article is based	21	*“This study did not receive external grant funding.”* (p. 21)

*Give information separately for cases and controls in case-control studies and, if applicable, for exposed and unexposed groups in cohort and cross-sectional studies.

Note: An Explanation and Elaboration article discusses each checklist item and gives methodological background and published examples of transparent reporting. The STROBE checklist is best used in conjunction with this article (freely available on the Web sites of PLoS Medicine at http://www.plosmedicine.org/, Annals of Internal Medicine at http://www.annals.org/, and Epidemiology at http://www.epidem.com/). Information on the STROBE Initiative is available at www.strobe-statement.org.
